# Towards Precision Medicine in Sinonasal Tumors: Low-Dimensional Radiomic Signature Extraction from MRI

**DOI:** 10.3390/diagnostics15131675

**Published:** 2025-06-30

**Authors:** Riccardo Biondi, Giacomo Gravante, Daniel Remondini, Sara Peluso, Serena Cominetti, Francesco D’Amore, Maurizio Bignami, Alberto Daniele Arosio, Nico Curti

**Affiliations:** 1 IRCCS Istituto delle Scienze Neurologiche di Bologna, Data Science and Bioinformatics Laboratory, 40139 Bologna, Italy; riccardo.biondi7@unibo.it; 2 Division of Otorhinolaryngology, Department of Biotechnology and Life Sciences, University of Insubria, Ospedale di Circolo, 21100 Varese, Italy; giacomo.gravante1@gmail.com (G.G.); cominetti53@gmail.com (S.C.); maurizio.bignami@uninsubria.it (M.B.); albertodaniele.arosio@gmail.com (A.D.A.); 3 Department of Physics and Astronomy, University of Bologna, 40127 Bologna, Italy; nico.curti2@unibo.it; 4 INFN, 40127 Bologna, Italy; 5 Department of Medical and Surgical Sciences, University of Bologna, 40138 Bologna, Italy; sara.peluso5@unibo.it; 6 IRCCS Azienda Ospedaliero Universitaria di Bologna, 40138 Bologna, Italy; 7 Department of Neuroradiology, University of Insubria, Ospedale di Circolo, 21100 Varese, Italy; francesco.damore@asst-settelaghi.it; 8 Head and Neck Surgery & Forensic Dissection Research Center (HNS&FDRc), Department of Biotechnology and Life Sciences, University of Insubria, 21100 Varese, Italy

**Keywords:** radiomic, medical image analysis, feature selection, machine learning, otorhinolaryngology

## Abstract

**Background:** Sinonasal tumors are rare, accounting for 3–5% of head and neck neoplasms. Machine learning (ML) and radiomics have shown promise in tumor classification, but current models lack detailed morphological and textural characterization. **Methods:** This study analyzed MRI data from 145 patients (76 malignant and 69 benign) across multiple centers. Radiomic features were extracted from T1-weighted (T1-w) images with contrast and T2-weighted (T2-w) images based on manually annotated tumor volumes. A dedicated ML pipeline assessed the effectiveness of different radiomic features and their integration with clinical variables. The DNetPRO algorithm was used to extract signatures combining radiomic and clinical data. **Results:** The results showed that ML classification using both data types achieved a median Matthews Correlation Coefficient (MCC) of 0.60 ± 0.07. The best-performing DNetPRO models reached an MCC of 0.73 (T1-w + T2-w) and 0.61 (T1-w only). Key clinical features included symptoms and tumor size, while radiomic features provided additional diagnostic insights, particularly regarding gray-level distribution in T2-w and texture complexity in T1-w images. **Conclusions:** Despite its potential, ML-based radiomics faces challenges in clinical adoption due to data variability and model diversity. Standardization and interpretability are crucial for reliability. The DNetPRO approach helps explain feature importance and relationships, reinforcing the clinical relevance of integrating radiomic and clinical data for sinonasal tumor classification.

## 1. Introduction

Sinonasal tumors are rare conditions that include both benign and malignant entities. Malignant tumors account for 3–5% of all head and neck neoplasms and often carry a poor prognosis, primarily due to late diagnosis [1,2,3]. Although benign tumors typically exhibit non-aggressive behavior, they can appear at advanced clinical stages and, like malignant tumors, produce nonspecific symptoms. Moreover, certain benign tumors have the potential to recur or even to transform into malignancies [4]. Distinguishing between malignant (cancerous) and benign (non-cancerous) tumors is crucial, as it significantly impacts on patient management, prognosis, and treatment strategies [5,6,7].

The initial diagnostic approach is generally similar for both benign and malignant tumors. It involves a comprehensive physical examination, including nasal endoscopy, imaging, and tissue biopsy [5,6]. Radiological evaluations are essential for diagnosing, staging, and determining the feasibility of tumor resection. Both Computed Tomography (CT) and Magnetic Resonance Imaging (MRI) are indispensable for devising an appropriate treatment plan [8].

CT scans, while helpful, provide limited insight into specific tumor patterns, with exceptions such as fibro-osseous lesions like osteomas, fibrous dysplasia, and ossifying fibromas [9]. Additionally, CT scans may overestimate disease extent, as they cannot reliably differentiate between tumors, inflamed mucosa, or retained secretions [10,11]. To better delineate tumor patterns, assess surrounding soft tissues, and evaluate orbital or intracranial involvement, MRI is often superior compared to CT, due to its ability to manipulate contrast between different tissues through techniques such as fat suppression, diffusion weighting, and many others. Imaging features on conventional MR sequences, such as T_2_-weighted (T_2_-w) and T_1_-weighted (T_1_-w) images (with and without gadolinium-based contrast agent, GBCA), may predict tumor histology before biopsy [8,10].

However, MRI diagnostic accuracy is limited by spatial resolution and relies on the operator’s expertise, introducing an element of subjectivity. Furthermore, the morphological features of benign and malignant sinonasal tumors often overlap, complicating their differentiation [12]. As a result, traditional imaging techniques only scratch the surface of the diagnostic potential they could provide.

The emergence of artificial intelligence (AI), machine learning (ML), and radiomics holds promise for addressing these limitations [13]. These technologies can extract hidden and otherwise inaccessible radiological information, potentially improving diagnostic precision and accelerating the pathway to a final diagnosis. This advancement could significantly enhance treatment planning and prognosis [12,14,15,16,17,18,19,20,21,22].

The aim of this study was to analyze the potential of utilizing a radiomic pipeline in the analysis of conventional MRI scans to determine the classification of sinonasal and skull base neoplasms as either benign or malignant. Clinical and radiomic variables will be evaluated and selected to possibly improve the discrimination model. A dedicated ML pipeline for the classification of malignant and benign tumors was developed. The analyses were performed using both radiomic and clinical features, proving the effectiveness of their synergy in determining the most informative subset of variables. Emphasis was given to the feature selection strategy employed for data integration, which represents a hot topic in the current radiomic literature. A hybrid signature of clinical and radiomic features will be proposed, customizing the DNetPRO algorithm [23] for this purpose [24]. The classification performances of the DNetPRO signature were compared to other standard ML approaches. The possibility to inspect the elements of the network signature extracted by the DNetPRO algorithm also provided explainable results associated with the numerical performance of the model. To the authors’ knowledge, this is the first application of the DNetPRO approach to this kind of tumor and the second on radiomic feature selection. This technique represents a technical innovation in this field, allowing the depiction of the most important features as well as their relation.

## 2. Materials and Methods

### 2.1. Study Population and Patient Selection

A retrospective study was conducted using data from patients referred to two Italian tertiary-care referral centers for sinonasal and skull base tumors, namely “Ospedale di Circolo e Fondazione Macchi” in Varese and “Ospedale Sant’Anna” in Como. The reference period spanned from 1 January 2011, to 1 April 2024. The inclusion criteria were as follows: (i) histopathologically diagnosed sinonasal or skull base tumors; (ii) primary or recurrent tumors; (iii) MRI examination performed no more than 4 weeks before the intended treatment; (iv) MRI studies including contrast-enhanced T_1_-w images (CE T_1_-w in the following) and axial T_2_-w images; (v) image acquisition on 1.5T scanners. The exclusion criteria were as follows: (a) incomplete or unavailable clinical or radiological data; or (b) MR sequences with a signal-to-noise ratio ≤ 1.0. All tumors were categorized as either benign or malignant based on histopathological analysis and according to the most recent World Health Organization (WHO) classification [25]. This study was approved by the Institutional Review Board of the hospitals (Insubria Board of Ethics, approval number 0033025/2015, approval date: 7 July 2015), and written informed consent was waived.

### 2.2. Image Acquisition and Segmentation

All participants underwent an MRI examination protocol that included at least the following sequences: fast spin echo (FSE) T_1_-w, FSE fat-saturated T_2_-w, and CE FSE T_1_-w. The latter sequence was acquired following intravenous administration of gadolinium (gadopentetate dimeglumin, Magnevist; Bayer 0.2 mL/kg or gadobutrol, Gadovist; Bayer 0.1 mL/kg). All the scans were conducted using two different 1.5 T MR scanners (Philips Achieva, in Varese Hospital; GE signa in Como Hospital), all of them with 1.5 T magnetic field strength. MR images were reviewed by an experienced head and neck radiologist to confirm the visual quality, readability, and adequacy for the following analytic phases.

Imaging alignment and 3D volume of interest (VOI) semi-automatic segmentation were performed on CE T_1_-w and T_2_-w using ITKSNAP software (version 3.8.0) [26]. Two additional VOI semi-automatic segmentations and an alignment review were performed for intra-observer agreement assessment by two expert radiologists. Subsequently, the processed alignment and semi-automatic segmentation were independently reviewed for inter-observer agreement reliability. Intra-/inter-observer disagreements were resolved by consensus. Quantitative inter-/intra-observer variability was assessed using the Dice similarity coefficient (DSC), which yielded a value of 0.90.

### 2.3. Clinical and Radiological Variables Selection

The population analyzed in the current study includes 145 patients, split into 76 (52%) malignant tumors and 69 (48%) benign ones. Patient demographics, tumor details, and clinical characteristics were recorded in a dedicated database and explained in Table 1. For some patients, the whole set of characteristics was not available, leading to some missing values (i.e., for tumor size, bone involvement, perineural spread, gross adjacent involvement, and epicenter).

Clinical information included age, sex, risk factors, symptoms, and tumor side. More specifically, symptoms were divided into class 1, asymptomatic or nonspecific (nasal obstruction, anosmia, headache, and epiphora), and class 2, red flags (persistent epistaxis, visual impairment, diplopia, proptosis, face pain, ocular movement pain, and face or palatal swelling). Imaging features included tumor size (more or less than 5 cm), T_2_-w low-signal (more or less than 50% of tumor), margins (well- or ill-defined), cystic component (yes/no), necrosis (more or less than 10% of tumor), septations (yes/no), bone involvement (yes/no), pattern of enhancement (heterogeneous or homogeneous), perineural spread (yes/no), midline crossing (yes/no), epicenter (nasal cavity vs. ethmoid sinus vs. maxillary sinus vs. frontal sinus vs. sphenoid sinus), and gross adjacent site involvement (yes/no) of at least one of the following: orbit, brain, nasolacrimal drainage system, palate, or skin.

The statistical analysis of clinical and demographic characteristics was performed, transforming the values into categorical variables. The categorical association between clinical variables and tumor outcomes was assessed using a Cramer’s V statistic [27] (using Bergsma Wicher correction [28]). The association test with tumor outcomes indicated a lower significance for all the considered variables except for gross adjacent site involvement (0.38), symptoms (0.41), and tumor Size (0.38).

### 2.4. Radiomic Features Extraction

For each lesion volume mask, a standard set of radiomic features was extracted from the lesion volume mask VOI on both the CE T_1_-w and T_2_-w images. The extracted features include first-order statistics, 3D shape-based scores, gray level co-occurrence matrix, gray level run length matrix, gray level size zone matrix, neighboring gray tone difference matrix, and gray level dependence matrix. For the sake of simplicity, we will label this set of variables as Original in the following sections. Associated with this first set of variables, an analogous amount of information was extracted by the images transformed using Laplacian of Gaussian (LoG) (with sigma of 0.5, 1.0, 1.5 and 2.0 mm) and wavelet transform (with 10 as the bin width parameter). For the sake of clarity, we will refer to these two sets of radiomic features as LoG and Wavelet, respectively. At the end of the extraction, each patient was characterized by a total of 1224 radiomic variables, split into the highlighted Original, LoG, and Wavelet for each image modality (CE T_1_-w or T_2_-w). The radiomic feature extraction was performed using the pyRadiomics [29] Python package (v3.1.0, accessed on 24 March 2024).

### 2.5. Radiomic Feature Embedding

A preliminary analysis of the radiomic features was assessed using a dimensionality reduction procedure. The variables were preliminarily standardized according to their median and interquartile values. The PaCMAP [30] dimensionality reduction algorithm was used to project the high-dimensional feature space onto a 2D space, keeping the hospital center and the tumor types as reference for the clustering enrichment. The aim of this analysis was to ensure the presence of possible batch effects in the data and/or the possibility to identify structures in the embedding space representation able to carry out information about the nature of the tumor type in a complete unsupervised framework.

### 2.6. Radiomic Feature Selection

The automated identification of malignant and benign tumors could be interpreted as a binary classification procedure. To this purpose, a dedicated ML pipeline was developed for the identification of the most informative features and outcome classification. Due to the large number of radiomic variables and the intrinsic co-linearity of the information, an accurate feature selection procedure represents a mandatory task for the noise reduction and final interpretability of the results. In this work, we adapted the DNetPRO [23] algorithm to manage high-dimensional radiomic feature space [24]. The original version of the algorithm was developed to handle gene expression data, typically characterized by a high number of features compared to the low available sample. An analogous behavior could be found also in radiomic data analysis, in which the redundancy of radiomic variables could lead to difficulties in the identification of a small interpretable subset of them. The possibility to identify a low dimensional set of features—a signature—described by a network relationship between them could facilitate the interpretability of the results, also facilitating the explainability of the classification model. For this purpose, we adapted the DNetPRO algorithm inserting linear Support Vector Machine (SVM) (in the couple evaluation step) and Penalized Logistic Regression models (in the best signature identification step) for the evaluation of feature pairs and filtering of the resulting signature. The results without the insertion of the DNetPRO procedure, i.e., applying an SVM classifier to all variables together, were used as a benchmark and discussed in terms of classification performance and model explainability.

### 2.7. Machine Learning Pipeline

**Figure 1:**
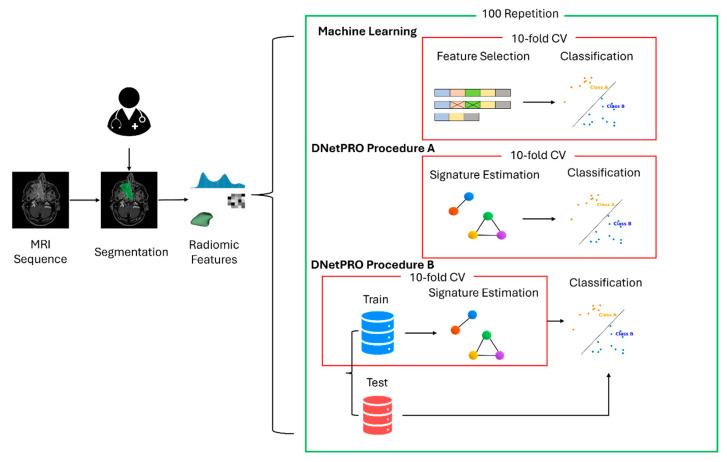
Scheme of the proposed ML pipelines. Outline of the proposed pipelines. Starting from the MR images, an expert clinician performs the manual identification and segmentation of the tumor volume on which the radiomic features were extracted. The data were split into train–test (and validation) according to a cross-validation scheme, and the different feature selection approaches (standard machine learning or DNetPRO) were applied to feed the final classification model.

Following the same scheme proposed for the feature embedding analysis, a dedicated ML pipeline for the classification of tumor types was developed independently for clinical and radiomic features. The intrinsic different but complementary nature of these two sources of information was considered, comparing the classification performances with a final model integrating both sources. In all the simulations, the developed ML pipeline involved a preprocessing step for the standardization of the values according to mean and standard deviation, followed by a feature selection procedure (DNetPRO or SVM classifier [31]), which led to filtering only the variables considered by a Penalized Logistic Regression model used for the binary classification. The data were split into training and test sets using a 10-fold cross-validation procedure, tuning the model parameters on the training set and evaluating the obtained performance on the related test set. The use of the DNetPRO algorithm was tested following both *procedure A* and *procedure B* (according to the nomenclature described in the original paper). Due to the low number of samples involved in the current study, we applied DNetPRO *procedure B* using a 3-step hold-out partitioning of the dataset, iterating the procedure 3 times. The schematic representation of the proposed pipelines is reported in Figure 1.

All these procedures were performed on features extracted from CE T_1_-w and T_2_-w separately and in combination. For each of the three classifications, a reduced set of radiomic features was used to remove redundant information and to focus on the information gain that could be achieved by integrating radiomic and clinical variables.

We identified the best set of radiomic features among the Original, LoG, and Wavelet categories by testing the performance of an SVM classifier over them separately. The evaluation of the feature category performance was carried out by repeating a stratified 10-fold cross-validation 100 times and employing the median and interquartile range (IQR) as a performance indicator. The performances were quantified using the Matthews Correlation Coefficient (MCC) score, aiming to prevent possible unbalanced results between the two classes. In this way, it was possible to consider the bias of the validation dataset and the different performances that could be achieved by different parameter initialization.

Once the most informative radiomic feature category was identified, it was analyzed using the DNetPRO A and B procedures. The different approaches (SVM and DNetPRO) were compared considering the distribution of the results (after 100 repeated stratified 10-fold cross-validation iterations) achieved on the same category of radiomic features, using the Wilcoxon test. Finally, the DNetPRO signature and the most relevant features for the classic radiomic approach were analyzed.

## 3. Results

### 3.1. Batch Effect Monitoring

**Figure 2:**
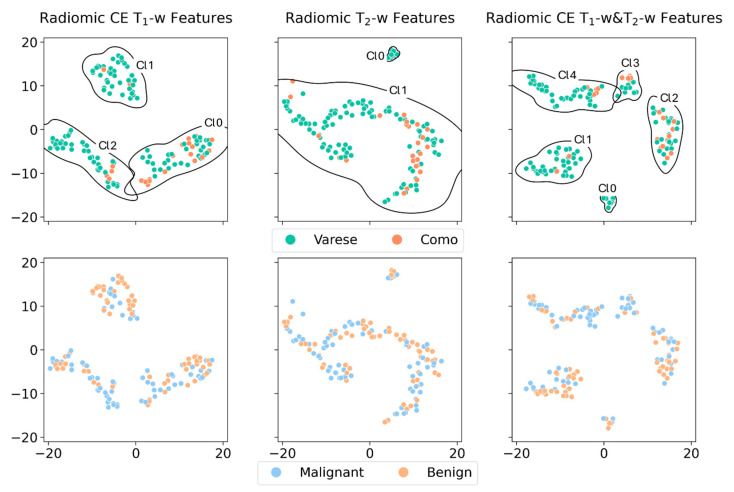
Radiomic embedding analysis. PacMAP projections of the radiomic data extracted from CE T_1_-w, T_2_-w, and CE T_1_-w and T_2_-w images. For each combination of features, we colored the data points according to the center of provenance (Varese and Como, first row) and in relation to the cancer type (Malignant and Benign, second row). The clustering of the points was performed using the HDBSCAN algorithm: for the sake of readability, the cluster labels were reported only in the upper row, associated with the kernel density estimations of the cluster boundaries.

We applied HDBSCAN, an unsupervised clustering method, to the 2D PaCMAP projections of CE T1-weighted, T2-weighted, and combined CE T1-weighted and T2-weighted MRI data. The resulting clusters were labeled according to the hospital where the MRI was performed (Varese or Como) or by tumor type (benign or malignant). This allowed us to check for potential batch effects linked to the imaging center, or for any natural grouping by tumor type, using Fisher’s exact test. Figure 2 shows the PaCMAP embeddings along with the HDBSCAN clusters. While all three datasets showed some visible clustering, only a few clusters had statistically significant associations with either the hospital or tumor type. For the imaging center, significant differences were observed in CE T1-weighted data between clusters Cl0 and Cl1 (*p* = 0.0003) and between Cl0 and Cl2 (*p* = 0.004), as shown in the top-left of Figure 2. In the combined T1 and T2 dataset, differences were also found between Cl1 and Cl2 (*p* = 0.006), and between Cl1 and Cl3 (*p* = 0.004) (Figure 2, top right). As for tumor type, only the CE T1-weighted data showed a significant difference between Cl1 and Cl2 (Figure 2, bottom left). No significant associations were seen with the T2-weighted data for either the hospital or tumor labels. Finally, when considering both hospital and tumor type together, the Fisher test showed no significant result (*p* = 1.0).

### 3.2. Radiomic Features’ Informative Power

Table 2 shows the median MCC along with one IQR as error, based on 100 runs of the classification pipeline using stratified data splits. The models were trained separately on features from CE T1-weighted, T2-weighted, and combined CE T1- and T2-weighted datasets. The machine learning pipeline included three steps: first, standard scaling; then feature selection using a linear-kernel SVM; and finally, classification with a ridge classifier based on the selected features. We used the median and IQR to report performance rather than the mean and standard deviation, as we could not assume a specific distribution shape for the MCC scores.

By looking at Table 2, it is possible to depict the most informative set of radiomic features (Original, LoG, or Wavelet) for each considered imaging modality. This contribution was used to select a single radiomic features category to use in the DNetPRO feature selection algorithm to reduce feature redundancy and computational requirement. The selected feature sets are the LoG for CE T_1_-w MRI sequences, the Original for the T_2_-w MRI sequences, and the Wavelet for the combination of the two. These radiomic feature sets were chosen as the ones maximizing the median of MCC score distribution.

**Figure 3:**
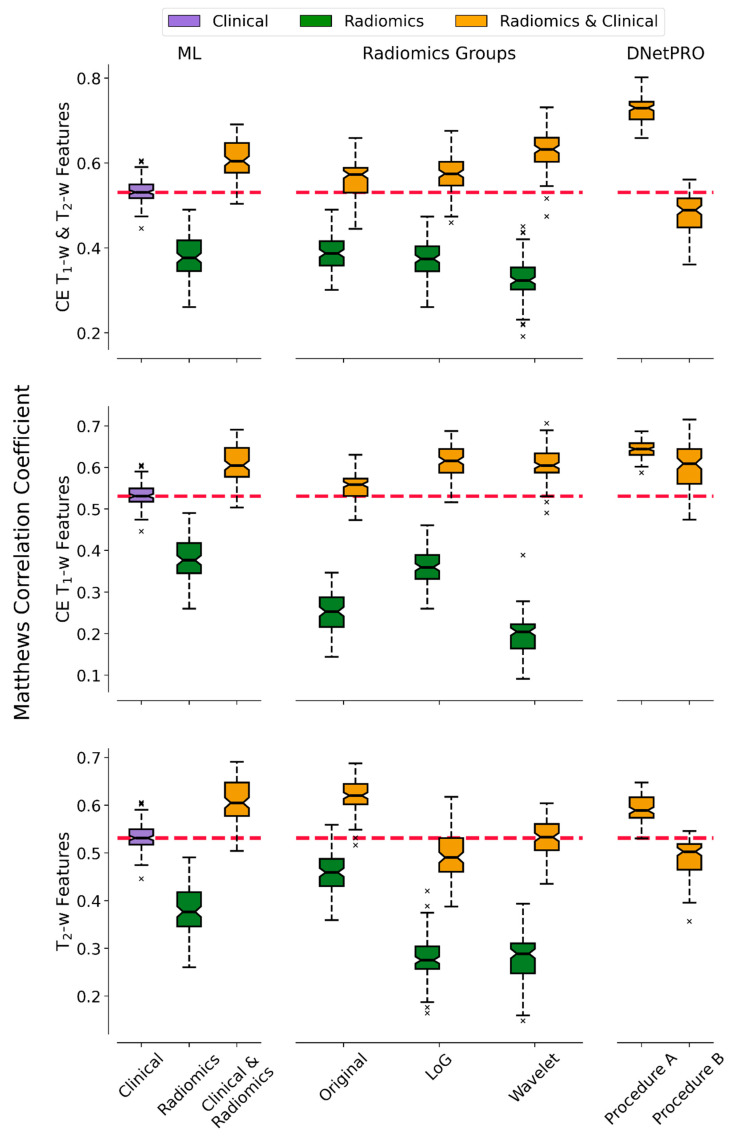
Distributions of performances stratified according to the different analyses. Starting from the top, we reported the results obtained by considering the combination of CE T_1_-w and T_2_-w radiomic features, the CE T_1_-w-only subset, and the T_2_-w-only subset. In the left column, we reported the performances obtained by the application of a standard ML pipeline on the clinical, radiomics, and clinical and radiomics combinations of data. In the central column of the figure, we reported the performances obtained considering each radiomic group of variables individually. The right column shows the performance obtained by the application of the DNetPRO algorithm, split in relation to the two possible procedures. For each plot, we highlighted with a dashed red line the median score obtained by considering only the clinical values, keeping it as reference for the radiomic contribution in the classification task.

In Figure 3 the MCC performance distributions of the classification pipelines are reported, obtained by using the DNetPRO approach or the SVM classifier over the different combinations of radiomic and clinical features. In this analysis we aimed to determine the informative contribution of each radiomic feature category and to measure the effectiveness of their integration in the classification task.

**Figure 4:**
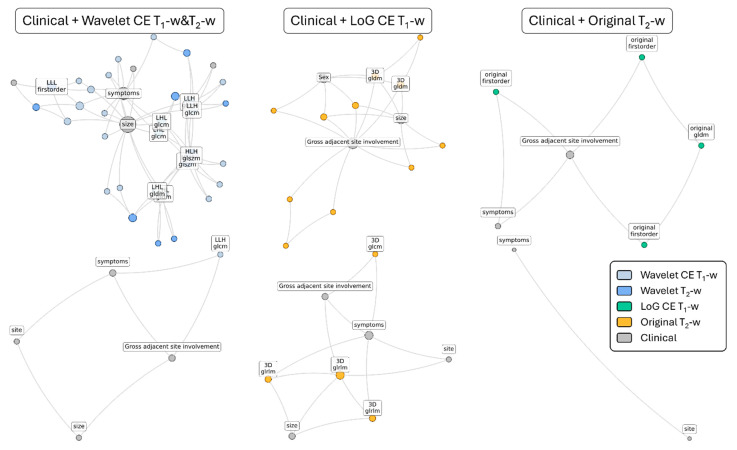
DNetPRO best signatures. Network representations of the best signatures identified by the DNetPRO application, according to procedure A (first row) and procedure B (second row), stratified according to the radiomic groups of features (Wavelet, LoG, and Original). The links between features consider positive/negative synergies of their mutual informative power in the classification task. The node size is represented proportionally to the degree of centrality in the network structure, identifying their importance and ability to cooperate with the other features of the signature network. For the sake of readability, in each plot we highlighted the names of the most central features; the full list of features in each signature and the corresponding network structure are reported in the Supplementary Materials.

In Figure 4 the best signatures identified by each DNetPRO pipeline (*procedure A* or *procedure B*) on each set of features (clinical + Wavelet, clinical + CE T_1_-w LoG, and clinical + T_2_-w Original) are reported. The use of the DNetPRO as the feature selection method allows us to monitor the finer-grain interaction between the features in the graph scheme, allowing explanations and interpretation of their mutual interaction and effectiveness in the classification. A detailed description of the features involved in the signature definitions can be found in the Supplementary Materials. Considering both Figure 4 and the detailed signature description in the Supplementary Materials, it is evident how the “gross adjacent site involvement”, “tumor size”, and “symptoms” clinical features play a central role in all the signatures, serving as network hubs. The selected radiomic features generally do not constitute high central nodes; rather, they complement the clinical features by integrating their information. It is worth noting that some of the selected radiomic features appear in pairs: for instance, the “Sum Average” and the “Joint Average” extracted from the gray level co-occurrence matrix always appear coupled, also sharing the same connection. Another example includes the “Low-“ and “High-Gray Level Emphasis” from the gray level distance matrix and the “Interquartile Range” and “Robust Mean Absolute Deviation”, extracted from the gray level histogram. All these features are highly correlated or anti-correlated, reflecting the redundant nature of radiomic features, as they carry the same information about the analyzed VOI. Finally, the most important features found in the signatures are the ones containing the same information for each MRI modality, regardless of the pipeline configuration. For the T_2_-w MRI sequences, the principal emerging aspect is the range of the gray level, depicted by the “Interquartile Range” and the “Robust Mean Absolute Deviation” features. In the case of features extracted from the wavelet-transformed image, a gain of importance is observed for the distribution of zones with high gray level in the case of wavelet transforms. For the Wavelet case, most of the features are derived from the image encoded with a low pass filter, i.e., from a denoised version of the original image. Finally, for the CE T_1_-w MRI sequences, the most relevant features describe the fineness or the coarseness of the VOI texture. Moreover, the presence or absence of large homogeneous areas, in terms of gray level, is considered by the “Size Zone Non-Uniformity Normalized” feature, which integrates the information provided by the “Joint Average” feature. Finally, information about the gray level intensity and its distribution are considered by the “Median” and “Skewness” of the gray level histogram.

To summarize, the PaCMAP application allowed us to estimate eventual batch effects. The most relevant radiomic features were identified for each MRI modality. From the DNetPRO feature selection approach, it was possible to observe how tumor size and symptoms consistently act as central hubs in classification signatures.

## 4. Discussion

The application of radiomics and ML to MRI in the analysis of sinonasal tumors has demonstrated remarkable potential, achieving significantly higher diagnostic accuracy compared to traditional methods [13]. However, despite these advancements, challenges persist in translating these tools into routine clinical practice. One key limitation lies in the variability of results across studies, driven by differences in data quality, sample size, and the diversity of AI models used. Moreover, the “black box” nature of many AI algorithms raises concerns about the interpretability and clinical reliability of the results.

To ensure that these innovative approaches become practical tools in everyday diagnostics, it is essential to prioritize the development of models that not only achieve high accuracy but also offer interpretable outcomes. This interpretability is crucial for fostering trust among clinicians and for guiding informed decision-making in patient care. Additionally, standardizing radiomic feature extraction, model validation, and reporting practices will be necessary to enhance reproducibility and comparability across studies. In our analysis, we tried to overcome this issue, providing a set of explainable, low-dimensional, and interpretable overviews of the most informative radiomic features.

Starting with the cluster analysis of the PaCMAP space, a partial stratification related to the hospital center is observed, possibly related to different acquisition devices and procedures. But since the distribution of malignant and benign samples is very balanced in the two centers, this suggests that the batch effect provided by the different centers is mitigated; thus, the findings from the ML analysis should not be significantly biased.

Performance analysis of the ML pipeline demonstrates that radiomic features alone have less predictive power than clinical features alone. However, when combined, the classifier’s performance improves, suggesting that radiomic features provide complementary information to clinical features.

Analyzing separately the contribution of individual categories of the radiomic features, stratified by the MRI modality from which they are extracted (CE T_1_-w, T_2_-w, and CE T_1_-w and T_2_-w) and by filters applied to the image itself (Original, LoG, and Wavelet), it was possible to observe how different features impact on classification. Comparing the classification results of DNetPRO and SVM, it is possible to observe how some results improve by considering only a category of radiomic features. That is the case of the Wavelet features from both the CE T_1_-w and T_2_-w sequences, the LoG features from the CE T_1_-w MRI sequence only, and the Original features for the T_2_-w sequence only, possibly because radiomic features provide redundant information, reducing the performances of the ML models.

Moreover, it is possible to explain the meaning of the set of most informative features selected for each image modality. The LoG extracts information about edges and blobs of a certain scale (specified by the sigma parameter); since the CE T_1_-w MRI sequence provides precise structural and morphological information, we can surmise that the LoG filter and the radiomic features are able to retrieve precise information about the tumor border and its internal structure (in term of uniformity or pattern complexity), also allowing the consideration of the adjacent inflamed mucosa. Since the T_2_-w MRI sequence can better visualize the tumor, the informative power of the radiomic features extracted from the original image (Original) is prominent, since it characterizes the texture and image appearance. Finally, radiomic features extracted from CE T_1_-w and T_2_-w images after wavelet transform are the most informative set when combining the two image modalities. This is likely due to the application of low-pass and high-pass filters in the wavelet transform computation, which resulted in images carrying similar information to the original and LoG-filtered images. The low-pass filter produces a denoised version of the original image, preserving the same information. In contrast, the high-pass filter enhances the edges, yielding results like those from the LoG filter. Based on the identified signature, most of the selected T_2_-w MRI features are derived from the image filtered with the low-pass filter. For the features selected from the CE T_1_-w images, they are extracted after applying the high-pass filter in at least one direction. This behavior supports the hypothesis that the Wavelet features contain information from both the Original and LoG categories.

Considering the DNetPRO approach, it was possible to observe that *procedure A* outperforms *procedure B* every time, possibly due to the low number of samples, which makes splitting the dataset into three parts is more difficult. The small number of patients represents an intrinsic limit of the current study, further penalizing the approaches in which a greater split of the data is required. Analogous results were also found and discussed in the work of Curti et al. [23], in which the authors emphasized the importance of a correct approach in terms of ML pipeline, considering the *procedure A* structure valid for a fair comparison of the state-of-the-art literature.

According to the Wilcoxon one-sided test, the DNetPRO *procedure B* performance is lower than ML in the T2 (*p* = $2\times 10^{-18}$) and T1 + T2 (*p* = $2\times 10^{-18}$) cases; for the T1 case, the test was not significant (*p* = 0.18). However, the DNetPRO signature provides valuable information about feature subsets, their relation, and their relevance for classification.

Analyzing the best clinical–radiological signatures selected by the model, the significance of the “Symptoms” and “Tumor Size” variables was remarkable. This highlighted the role of the so-called “red flags” in malignant tumors as an alarm for a potentially aggressive disease. Both variables appeared to be relevant, particularly when correlated with specific radiomic parameters. Other variables considered by the model included the gross evident involvement of adjacent sites and the tumor epicenter.

This also mirrored the clinical practice, as malignant tumors are more likely to involve structures such as the orbit, brain, lacrimal pathway, palate, and skin. Despite the absence of other clinical–radiological variables among the top signatures, their relevance was not dismissed but rather relegated to a secondary role. The model considered these variables less consistent when associated with the selected radiomic indicators.

The DNetPRO approach enhances the understanding and interpretability of the signature and the relationships between the selected features. Upon studying the signature, it becomes evident that radiomic features do not play a central role in classification; instead, they integrate the information provided by the clinical features, contributing to more accurate classification results. As expected, redundant radiomic features are also present. Redundancy is highlighted by the fact that these features are related within the same adjacency matrix, sharing interactions with other covariates. Finally, an interpretation of the radiomic component of the signatures can be proposed. The significance of gray level distribution and histogram dispersion for features extracted from the T_2_-w MRI sequence may reflect the degree of internal heterogeneity within the tumor. Malignant tumors often exhibit such heterogeneities, including areas of necrosis or high cellular density, which are less commonly observed in benign tumors, typically characterized by greater uniformity. Moreover, the presence of complex texture patterns in the CE T_1_-w image may be indicative of malignant tumors, which frequently display varied and irregular structural features, such as calcifications or hemorrhages. In contrast, large uniform areas are more commonly associated with benign tumors, such as cysts or fibrous lesions.

The proposed model, by integrating clinical and radiomic features, can significantly enhance the routine clinical workflow in the evaluation of sinonasal tumors. It has the potential to assist clinicians in expediting the diagnostic process by providing probabilistic discrimination between benign and malignant lesions prior to the biopsy. This can reduce diagnostic delays and help prioritize cases that require urgent attention, thus optimizing clinical workflow and resource allocation. Moreover, this model could especially assist non-referral or peripheral centers that may lack specialized expertise in sinonasal tumors. It can also play a crucial role during patient follow-up, aiding in distinguishing between a true tumor recurrence and benign post-treatment changes or inflammatory lesions, particularly in anatomical regions where biopsy is challenging (e.g., frontal sinus). The integration of the radiomic component into MRI analysis software is a step that could further empower radiologists by providing real-time, data-driven insights when interpreting complex or equivocal imaging findings. This decision support system would enhance radiological accuracy and confidence, ultimately streamlining the diagnostic pathway.

## 5. Conclusions

The introduction of ML and radiomics is transforming sinonasal tumor diagnostics, addressing limitations of conventional MRI and radiologist interpretation. Despite progress, challenges like dataset size, class imbalance, and segmentation persist. Furthermore, a clear link between data and phenotypes of tumor volumes is still missing, highlighting the huge difficulty in radiomic explainability. The demand for explainable solutions and signatures represents a mandatory task for novel ML models to guarantee their application in clinical practice, requiring extra efforts in data analysis. In our application we tried to overcome this limitation by introducing an accurate feature selection step, showing how signatures identified using a network-based approach (DNetPRO) could facilitate this task.

The present study has several limitations that should be carefully considered when interpreting its findings and implications for clinical practice. Firstly, its retrospective design introduces inherent selection bias, relying on existing records which may not uniformly capture all relevant clinical and radiological variables. Additionally, the small sample size limits statistical power and generalizability, potentially affecting the model’s accuracy in differentiating between various histological types of sinonasal and skull base tumors. The relatively small dataset size does not allow the creation of an external test set, affecting the quantification of generalization capabilities. Furthermore, while semi-automated, the segmentation process involved manual steps, which may introduce bias and reduce reproducibility. Finally, the small dataset accounts could introduce bias from the two acquisition centers, but this effect was estimated and considered by the PaCMAP and clustering procedure.

However, the present study underscores ML potential in MRI analysis as a tool to aid clinicians in distinguishing between benign and malignant sinonasal tumors. From a multitude of radiomic features, we were able to identify essential signatures crucial for this differentiation, suggesting future integration into MRI software to support real-time diagnostic decision-making by radiologists.

## Figures and Tables

**Table 1:** Dataset description. Clinical and demographic characteristics of the patients included in the study. The age variable is described by the average and interquartile range. For all the other variables, we reported the percentage of samples and the corresponding number of patients.

Variable	Values	Amount
**Age** (mean, range)		54 (2–87)
**Male gender**		72% (104)
**Tumor type**	Malignant	52% (76)
	Benign	48% (69)
**Hospital center**	Varese	82% (119)
	Como	18% (26)
**Gross adjacent site involvement**	No	85% (123)
	Yes	15% (21)
**Symptoms**	Non-specific (asymptomatic, anosmia, headache, and epiphora)	46% (66)
	Red flags (persistent epistaxis, decreased vision, diplopia, proptosis, facial or eye pain, and swelling)	54% (79)
**Tumor size**	<5 cm	42% (61)
	≥5 cm	58% (83)
**Side**	Left	47% (68)
	Right	50% (73)
	Bilateral	3% (4)
**Margins**	Well-defined	33% (48)
	Ill-defined	67% (96)
**Patterns**	Homogeneous	21% (30)
	Inhomogeneous	79% (114)
**Necrosis**	≥10%	59% (85)
	<10%	41% (59)
**T_2_-w** **low-signal**	>50%	29% (42)
	≤50%	71% (102)
**Cystic component**	Yes	44% (64)
	No	56% (80)
**Septation**	Yes	8% (12)
	No	92% (132)
**Bone involvement**	Yes	57% (82)
	No	43% (62)
**Perineural spread**	Yes	16% (23)
	No	84% (121)
**Midline crossing**	Yes	35% (50)
	No	65% (94)
**Epicenter**	Ethmoid sinus	33.1% (47)
	Maxillary sinus	33.1% (47)
	Nasal cavity	25.4% (36)
	Frontal sinus	4.2% (6)
	Sphenoid sinus	4.2% (6)

**Table 2:** Radiomic feature performances. Performances were obtained by the application of a standard ML pipeline composed of feature standardization, feature selection, and classification model considering the three main groups of radiomic features integrated with the clinical one. The performances were quantified using MCC, expressed in terms of median ± one interquartile range. The performances were estimated according to 100 hold-out partitions of the available samples, training the model on 90% of the data and quantifying the scores on the remaining 10%, used as the test set.

	Original	LoG	Wavelet
**CE T_1_-w**	0.56 ± 0.04	**0.62 ± 0.06**	0.60 ± 0.05
**T_2_-w**	**0.62 ± 0.04**	0.49 ± 0.07	0.53 ± 0.06
**CE T_1_-w and T_2_-w**	0.57 ± 0.06	0.57 ± 0.06	**0.63 ± 0.06**

## Data Availability

The dataset used for the analyses and the code implemented for the reproducibility of the results are available from the corresponding author on reasonable request.

## Supplementary Materials

The following supporting information can be downloaded at: https://www.mdpi.com/article/10.3390/diagnostics15131675/s1, Figure S1: Representative examples of both T1-w and T2-w images; Table S1: DNetPRO signature extracted on Wavelet T1-w & T2-w + Clinical data with Procedure A; Table S2: DNetPRO signature extracted on LoG T1-w + Clinical data with Procedure A; Table S3: DNetPRO signature extracted on Original T2-w + Clinical data with Procedure A; Table S4: DNetPRO signature extracted on Wavelet T1-w & T2-w + Clinical data with Procedure B; Table S5: DNetPRO signature extracted on LoG T1-w + Clinical data with Procedure B; Table S6: DNetPRO signature extracted on Original T2-w + Clinical data with Procedure B.

## Abbreviations

The following abbreviations are used in this manuscript:

CT	Computer Tomography
MRI	Magnetic Resonance Imaging
T_1_-w	T_1_-weighted
T_2_-w	T_2_-weighted
GBCA	Gadolinium-based contrast agent
DNetPRO	Discriminant Analysis with Network Processing
AI	Artificial intelligence
ML	Machine learning
CE	Contrast-enhanced
FSE	Fast spin echo
VOI	Volume of interest
Original	Radiomic original features
LoG	Radiomic Laplacian of Gaussian features
Wavelet	Radiomic wavelet features
SVM	Support Vector Machine
IQR	Interquartile range
MCC	Matthew’s Correlation Coefficient

## References

[1] Abdou R., Baredes S. (2017). Population-Based Results in the Management of Sinonasal and Ventral Skull Base Malignancies. Otolaryngol. Clin. N. Am..

[2] Castelnuovo P., Turri-Zanoni M., Battaglia P., Antognoni P., Bossi P., Locatelli D. (2016). Sinonasal Malignancies of Anterior Skull Base. Otolaryngol. Clin. N. Am..

[3] Madani G., Beale T.J., Lund V.J. (2009). Imaging of Sinonasal Tumors. Semin. Ultrasound CT MRI.

[4] Luong T.T., Yan C.H. (2023). Benign Paranasal Sinus Tumors. Curr. Otorhinolaryngol. Rep..

[5] Bracigliano A., Tatangelo F., Perri F., Di Lorenzo G., Tafuto R., Ottaiano A., Clemente O., Barretta M.L., Losito N.S., Santorsola M. (2021). Malignant Sinonasal Tumors: Update on Histological and Clinical Management. Curr. Oncol..

[6] Ohshika S., Saruga T., Ogawa T., Ono H., Ishibashi Y. (2021). Distinction between Benign and Malignant Soft Tissue Tumors Based on an Ultrasonographic Evaluation of Vascularity and Elasticity. Oncol. Lett..

[7] Saito T., Nakayama M., Ohnishi K., Tanaka S., Nakamura M., Murakami M., Matsumoto S., Baba K., Fujii K., Mizumoto M. (2023). Proton Beam Therapy in Multimodal Treatment for Locally Advanced Squamous Cell Carcinoma of the Nasal Cavity and Paranasal Sinus. Radiat. Oncol..

[8] Kennedy D.W., Hwang P.H. (2012). Rhinology: Diseases of the Nose, Sinuses, and Skull Base.

[9] Ciniglio Appiani M., Verillaud B., Bresson D., Sauvaget E., Blancal J.-P., Guichard J.-P., Saint Maurice J.-P., Wassef M., Karligkiotis A., Kania R. (2015). Fibroma Ossificante Dei Seni Paranasali: Diagnosi e Management. Acta Otorhinolaryngol. Ital..

[10] Gomaa M.A., Hammad M.S., Abdelmoghny A., Elsherif A.M., Tawfik H.M. (2013). Magnetic Resonance Imaging versus Computed Tomography and Different Imaging Modalities in Evaluation of Sinonasal Neoplasms Diagnosed by Histopathology. Clin. Med. Insights Ear Nose Throat.

[11] Yousem D.M., Montone K.T. (1998). HEAD AND NECK LESIONS: Radiologic–Pathologic Correlations. Radiol. Clin. N. Am..

[12] Bi S., Zhang H., Wang H., Ge Y., Zhang P., Wang Z., Hao D. (2021). Radiomics Nomograms Based on Multi-Parametric MRI for Preoperative Differential Diagnosis of Malignant and Benign Sinonasal Tumors: A Two-Centre Study. Front. Oncol..

[13] Gravante G., Arosio A.D., Curti N., Biondi R., Berardi L., Gandolfi A., Turri-Zanoni M., Castelnuovo P., Remondini D., Bignami M. (2024). Artificial Intelligence and MRI in Sinonasal Tumors Discrimination: Where Do We Stand?. Eur. Arch. Oto-Rhino-Laryngol..

[14] Wang X.-Y., Yan F., Hao H., Wu J.-X., Chen Q.-H., Xian J.-F. (2015). Improved Performance in Differentiating Benign from Malignant Sinonasal Tumors Using Diffusion-Weighted Combined with Dynamic Contrast-Enhanced Magnetic Resonance Imaging. Chin. Med. J..

[15] El-Gerby K., El-Anwar M. (2017). Differentiating Benign from Malignant Sinonasal Lesions: Feasibility of Diffusion Weighted MRI. Int. Arch. Otorhinolaryngol..

[16] Jiang J.X., Tang Z.H., Zhong Y.F., Qiang J.W. (2017). Diffusion Kurtosis Imaging for Differentiating between the Benign and Malignant Sinonasal Lesions. J. Magn. Reson. Imaging.

[17] Van Timmeren J.E., Cester D., Tanadini-Lang S., Alkadhi H., Baessler B. (2020). Radiomics in Medical Imaging—“How-to” Guide and Critical Reflection. Insights Imaging.

[18] Zhang H., Wang H., Hao D., Ge Y., Wan G., Zhang J., Liu S., Zhang Y., Xu D. (2021). An MRI -Based Radiomic Nomogram for Discrimination Between Malignant and Benign Sinonasal Tumors. J. Magn. Reson. Imaging.

[19] Chen C., Qin Y., Chen H., Cheng J., He B., Wan Y., Zhu D., Gao F., Zhou X. (2022). Machine Learning to Differentiate Small Round Cell Malignant Tumors and Non-Small Round Cell Malignant Tumors of the Nasal and Paranasal Sinuses Using Apparent Diffusion Coefficient Values. Eur. Radiol..

[20] Bundschuh R.A., Dinges J., Neumann L., Seyfried M., Zsótér N., Papp L., Rosenberg R., Becker K., Astner S.T., Henninger M. (2014). Textural Parameters of Tumor Heterogeneity in ^18^F-FDG PET/CT for Therapy Response Assessment and Prognosis in Patients with Locally Advanced Rectal Cancer. J. Nucl. Med..

[21] Zhang Y., Lin N., Xiao H., Xin E., Sha Y. (2023). Differentiation of Sinonasal NKT from Diffuse Large B-Cell Lymphoma Using Machine Learning and MRI-Based Radiomics. J. Comput. Assist. Tomogr..

[22] Du L., Yuan Q., Han Q. (2023). A New Biomarker Combining Multimodal MRI Radiomics and Clinical Indicators for Differentiating Inverted Papilloma from Nasal Polyp Invaded the Olfactory Nerve Possibly. Front. Neurol..

[23] Curti N., Levi G., Giampieri E., Castellani G., Remondini D. (2022). A Network Approach for Low Dimensional Signatures from High Throughput Data. Sci. Rep..

[24] Dalmonte S., Cocozza M.A., Cuicchi D., Remondini D., Faggioni L., Castellucci P., Farolfi A., Fortunati E., Cappelli A., Biondi R. (2025). Identification of PET/CT Radiomic Signature for Classification of Locally Recurrent Rectal Cancer: A Network-Based Feature Selection Approach. Heliyon.

[25] Thompson L.D.R., Bishop J.A. (2022). Update from the 5th Edition of the World Health Organization Classification of Head and Neck Tumors: Nasal Cavity, Paranasal Sinuses and Skull Base. Head Neck Pathol..

[26] Yushkevich P.A., Piven J., Hazlett H.C., Smith R.G., Ho S., Gee J.C., Gerig G. (2006). User-Guided 3D Active Contour Segmentation of Anatomical Structures: Significantly Improved Efficiency and Reliability. NeuroImage.

[27] Cramér H. (1991). Mathematical Methods of Statistics.

[28] Bergsma W. (2013). A Bias-Correction for Cramér’s and Tschuprow’s. J. Korean Stat. Soc..

[29] van Griethuysen J.J.M., Fedorov A., Parmar C., Hosny A., Aucoin N., Narayan V., Beets-Tan R.G.H., Fillion-Robin J.-C., Pieper S., Aerts H.J.W.L. (2017). Computational Radiomics System to Decode the Radiographic Phenotype. Cancer Res..

[30] Wang Y., Huang H., Rudin C., Shaposhnik Y. (2020). Understanding How Dimension Reduction Tools Work: An Empirical Approach to Deciphering t-SNE, UMAP, TriMAP, and PaCMAP for Data Visualization. arXiv.

[31] Cortes C., Vapnik V. (1995). Support-Vector Networks. Mach. Learn..

