# Supplementary material for: Towards Precision Medicine in Sinonasal Tumors: Low-Dimensional Radiomic Signature Extraction from MRI
Source: Diagnostics (Basel). 2025 Jun 30;15(13):1675. doi: 10.3390/diagnostics15131675 (PMC12248528; doi:10.3390/diagnostics15131675)
Supplement: Supplementary file 1 [file diagnostics-15-01675-s001.zip › diagnostics-3680169-supplementary.pdf]

diagnostics

MDPI

Article

Towards Precision Medicine in Sinonasal Tumors: Low-Dimensional Radiomic Signature Extraction from MRI

Riccardo Biondi [1,†], Giacomo Gravante [2,†], Daniel Remondini [3,4,*], Sara Peluso [5,6], Serena Cominetti [2], Francesco D'Amore [7], Maurizio Bignami [2,8], Alberto Daniele Arosio [2,‡] and Nico Curti [3,4,‡]

[1] IRCCS Istituto delle Scienze Neurologiche di Bologna, Data Science and Bioinformatics Laboratory, 40139 Bologna, Italy; riccardo.biondi7@unibo.it
[2] Division of Otorhinolaryngology, Department of Biotechnology and Life Sciences, University of Insubria, Ospedale di Circolo, *21100* Varese, Italy; giacomo.gravante1@gmail.com (G.G.); cominetti53@gmail.com (S.C.); maurizio.bignami@uninsubria.it (M.B.); albertodaniele.arosio@gmail.com (A.D.A.)
[3] Department of Physics and Astronomy, University of Bologna, 40127 Bologna, Italy; nico.curti2@unibo.it
[4] INFN, 40127 Bologna, Italy
[5] Department of Medical and Surgical Sciences, University of Bologna, 40138 Bologna, Italy; sara.peluso5@unibo.it (S.P.)
[6] IRCCS Azienda Ospedaliero Universitaria di Bologna, 40138 Bologna, Italy
[7] Department of Neuroradiology, University of Insubria, Ospedale di Circolo, 21100 Varese, Italy; francesco.damore@asst-settelaghi.it
[8] Head and Neck Surgery & Forensic Dissection Research Center (HNS&FDRc), Department of Biotechnology and Life Sciences, University of Insubria, 21100 Varese, Italy
* Correspondence: daniel.remondini@unibo.it
† These authors contributed equally to this work.
‡ These authors contributed equally to this work.

Supplementary Materials

Extended Dataset Description

In the present study, we focus on two magnetic resonance imaging (MRI) modalities: T1-weighted and T2-weighted sequences, hereafter referred to as T1-w and T2-w, respectively. These imaging techniques are commonly used in clinical practice to evaluate soft tissue contrast and are particularly useful in the assessment of head and neck pathologies.

Supplementary Figure S1 illustrates representative examples of both T1-w and T2-w images. The figure displays coronal views of a patient diagnosed with a sinonasal tumor, showcasing both benign and malignant cases to highlight the radiological differences across tumor types.

Clinical information regarding tumor characteristics—specifically tumor size, gross adjacent site involvement, bone invasion, and perineural spread—is available for 144 out of the 145 patients included in the dataset. Additionally, information about the tumor epicenter is available for 142 patients. These data points are essential for accurate tumor staging and for guiding therapeutic decisions.

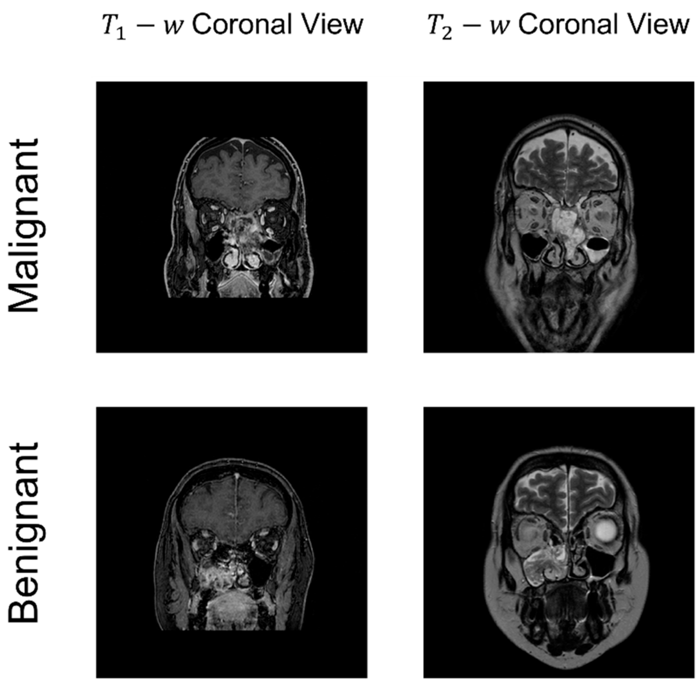

Supplementary Figure S1 From left to right: Coronal view of a patients affected by Sinonasal tumor of T_1-w and T_2-w sequences . From up to low, Coronal view of MRI sequences of patient affected by malignant and benignant sinonasal tumour.

DNetPRO signatures composition

In ***Supplementary Tables S1-S6*** we reported the detailed description of the best signatures extracted by the DNetPRO algorithm in the three different subsets of data. For each signature, the corresponding network structure is expressed in terms of adjacency list, preserving in this way the links between variables and allowing a complete reconstruction of the results presented in the original paper.

**Wavelet T_1-w & T_2-w Signature – Procedure A**			
**Variable Type**	**N**	**Feature Name**	**Adjacent List**
**Clinical**	0	Symptoms	3, 16, 22, 23, 24, 26, 27, 28, 33, 34
	1	Size	3, 15, 16, 17, 19, 20, 21, 22, 23, 25, 26, 27, 28, 31, 32, 33, 34
	2	Endo/CFR	5, 6
	3	Age	0, 1
	4	Sex	19, 20
**Radiomic T_2-w**	5	LLL firstorder InterquartileRange	2, 17, 16, 25, 26
	6	LLL firstorder RobustMeanAbsoluteDeviation	2, 16, 25
	7	HHL firstorder Range	19, 20
	8	HLH glszm SmallAreaLowGrayLevelEmphasis	19, 20, 24
	9	HLH glszm HighGrayLevelZoneEmphasis	18, 19, 20, 22, 23, 29, 30, 31, 32
	10	HLH glszm LowGrayLevelZoneEmphasis	18, 19, 20, 22, 23, 29, 30, 31, 32
	11	LLL glcm Autocorrelation	31, 32
	12	LLL gldm HighGrayLevelEmphasis	31, 32

	13	LLL glrlm HighGrayLevelRunEmphasis	15, 21, 31, 32
	14	LLL glrlm LowGrayLevelRunEmphasis	19, 20, 22, 23
**Radiomic T_1-w**	15	LHL firstorder Median	1, 13
	16	HLL firstorder Median	0, 1, 5, 6
	17	LLH glcm Autocorrelation	1, 4
	18	LLH glcm ClusterShade	9, 10
	19	LLH glcm JointAverage	1, 4, 7, 8, 9, 10, 14, 35
	20	LLH glcm SumAverage	1, 4, 7, 8, 9, 10, 14, 35
	21	LHL glcm ClusterShade	1, 13
	22	LHL glcm JointAverage	0, 1, 9, 10, 14
	23	LHL glcm SumAverage	0, 1, 9, 10, 14
	24	LHL glcm SumSquares	0, 8
	25	HLL glcm Autocorrelation	1, 5, 6
	26	HLL glcm ClusterShade	0, 1, 5
	27	HLL glcm JointAverage	0, 1
	28	HLL glcm SumAverage	0, 1
	29	LLH gldm HighGrayLevelEmphasis	9, 10
	30	LLH gldm LowGrayLevelEmphasis	9, 10
	31	LHL gldm HighGrayLevelEmphasis	1, 9, 10, 12, 13
	32	LHL gldm LowGrayLevelEmphasis	1, 9, 10, 12, 13
	33	HLL gldm HighGrayLevelEmphasis	0, 1
	34	HLL gldm LowGrayLevelEmphasis	0, 1
	35	HLH glszm SizeZoneNonUniformityNormalized	19, 20

Supplementary Table S1 | DNetPRO signature extracted on Wavelet T_1-w & T_2-w + Clinical data. *Radiomic and clinical network structure of the signature extracted by DNetPRO algorithm following procedure A. For each node/feature we describe the adjacency list of the network structure, following the enumeration of the nodes proposed in the second row.*

LoG T_1-w Signature – Procedure A			
**Variable Type**	**N**	**Feature Name**	**Adjacent List**
**Clinical**	0	Size	3, 4, 5, 6, 9, 10
	1	Endo/CFR	3, 4, 5, 6, 7, 8, 9, 10, 11
	2	Sex	3, 4, 5, 6, 7, 8, 9, 10, 11
**Radiomic T_1-w**	3	LoG 0.5 mm 3D glcm SumAverage	0, 1
	4	LoG 0.5 mm 3D glcm JointAverage	0, 1
	5	LoG 1.0 mm 3D glcm JointAverage	0, 1, 2
	6	LoG 1.0 mm 3D glcm SumAverage	0, 1, 2
	7	LoG 1.0 mm 3D glcm ClusterShade	1, 13
	8	LoG 1.0 mm 3D glcm Autocorrelation	1, 13
	9	LoG 1.0 mm 3D gldm HighGrayLevelEmphasis	0, 1, 2, 12
	10	LoG 1.0 mm 3D gldm LowGrayLevelEmphasis	0, 1, 2, 12
	11	LoG 1.5 mm 3D firstorder Median	1, 2
	12	LoG 1.5 mm 3D firstorder Skewness	9, 10
	13	LoG 2.0 mm 3D glrlm ShortRunEmphasis	7, 8

Supplementary Table S2 | DNetPRO signature extracted on LoG T_1-w + Clinical data. *Radiomic and clinical network structure of the signature extracted by DNetPRO algorithm following procedure A. For each node/feature we describe the adjacency list of the network structure, following the enumeration of the nodes proposed in the second row.*

Original T_2-w Signature – Procedure A			
**Variable Type**	**N**	**Feature Name**	**Adjacent List**
**Clinical**	0	Symptoms	1, 4
	1	Endo/CFR	0, 2, 3, 4
**Radiomic T_2-w**	2	firstorder InterquartileRange	1, 5
	3	firstorder RobustMeanAbsoluteDeviation	1, 5
	4	firstorder MeanAbsoluteDeviation	0, 1
	5	gldm SmallDependenceHighGrayLevelEmphasis	2, 3

Supplementary Table S3 | DNetPRO signature extracted on Original T_2-w + Clinical data. *Radiomic and clinical network structure of the signature extracted by DNetPRO algorithm following procedure A. For each node/feature we describe the adjacency list of the network structure, following the enumeration of the nodes proposed in the second row.*

Wavelet T_1-w & T_2-w Signature – Procedure B			
**Variable Type**	**N**	**Feature Name**	**Adjacent List**
**Clinical**	0	Symptoms	1, 2, 4
	1	Site	0, 3
	2	Endo/CFR	0, 3, 4
	3	Size	1, 2

**Radiomic T_1-w**	4	LLH glcm DifferenceAverage	0, 2

Supplementary Table S4 | DNetPRO signature extracted on Wavelet T_1-w & T_2-w + Clinical data. *Radiomic and clinical network structure of the signature extracted by DNetPRO algorithm following procedure B. For each node/feature we describe the adjacency list of the network structure, following the enumeration of the nodes proposed in the second row.*

LoG T_1-w Signature – Procedure B			
**Variable Type**	**N**	**Feature Name**	**Adjacent List**
**Clinical**	0	Symptoms	1, 2, 4, 6, 7
	1	Endo/CFR	0, 4, 5
	2	Site	0, 5
	3	Size	5, 6, 7
**Radiomic T_1-w**	4	LoG 0.5 mm 3D glcm Imc1	0, 1
	5	LoG 0.5 mm 3D glcm LowGrayLevelRunEmphasis	1, 2, 3, 6, 7
	6	LoG 0.5 mm 3D glrlm GrayLevelNonUniformity	0, 3, 5
	7	LoG 0.5 mm 3D glrlm GrayLevelVariance	0, 3, 5

Supplementary Table S5 | DNetPRO signature extracted on LoG T_1-w + Clinical data. *Radiomic and clinical network structure of the signature extracted by DNetPRO algorithm following procedure B. For each node/feature we describe the adjacency list of the network structure, following the enumeration of the nodes proposed in the second row.*

Original T_2-w Signature – Procedure B			
**Variable Type**	**N**	**Feature Name**	**Adjacent List**
**Clinical**	0	Symptoms	1
	1	Site	0

Supplementary Table S6 | DNetPRO signature extracted on Original T_2-w + Clinical data. *Radiomic and clinical network structure of the signature extracted by DNetPRO algorithm following procedure B. For each node/feature we describe the adjacency list of the network structure, following the enumeration of the nodes proposed in the second row.*

The dataset comprises 145 patients affected by sinonasal tumor, specifically, the target tumor types were Ameloblastoma, Intestinal type adenocarcinoma, Inverted Papilloma, ITAC recurrence, Juvenile Angiofibroma, Melanoma and Nasopharyngeal carcinoma; divided into benignant and malignant for classification purpose. As stated in the manuscript, the categorization was based on histopathological analysis, according to the World Health Organization (WHO) classification.
